# Radiation-induced cytochrome *c* release and the neuroprotective effects of the pan-caspase inhibitor z-VAD-fmk in the hypoglossal nucleus

**DOI:** 10.3892/etm.2013.1419

**Published:** 2013-11-20

**Authors:** JIANGUO LI, YAN WANG, LIQING DU, CHANG XU, JIA CAO, QIN WANG, QIANG LIU, FEIYUE FAN

**Affiliations:** 1 Tianjin Key Laboratory of Molecular Nuclear Medicine, Institute of Radiation Medicine, Chinese Academy of Medical Sciences and Peking Union Medical College, Nankai, Tianjin 300192, P.R. China; 2 Department of Human Anatomy, The Medical School of Inner Mongolia University for the Nationalities, Tongliao, Neimenggu 028041, P.R. China

**Keywords:** radiation, cytochrome *c*, hypoglossal nucleus, z-VAD-fmk, X-linked inhibitor of apoptosis protein, Smac, DIABLO

## Abstract

Numerous studies have demonstrated that neuronal cell death occurs via extrinsic (death receptors) and intrinsic (mitochondria) pathways. Radiation induces caspase activation fundamentally via the mitochondrial pathway. To investigate the role of caspase, a cell permeable pan-caspase inhibitor, z-VAD-fmk [N-benzyloxycarbonyl-Val-Ala-Asp(OMe)-fluoromethylketone], was used to investigate the effects of caspase blockade *in vivo* following irradiation. Adult male Sprague-Dawley rats (weight, 250–300 g) underwent irradiation at room temperature with a 4-Gy dose of radiation. Since z-VAD-fmk does not penetrate the blood-brain barrier, it was applied intracerebroventricularly via a bolus injection (0.2 μg/h for 1 h). Terminal deoxynucleotidyl transferase dUTP nick end-labeling (TUNEL) demonstrated that z-VAD-fmk reduced the numbers of TUNEL-positive cells within the hypoglossal nucleus, suggesting that intervention in the caspase cascade following radiation may have therapeutic applications. The caspase inhibitor z-VAD-fmk reduced the expression and activation of caspase-3, caspase-8 and caspase-9 in the irradiated rats, indicating that caspase may be a potential therapeutic target in the treatment of brain radiation injury. Treatment with z-VAD-fmk also reduced the appearance of cytochrome *c* within the cytosolic fraction following radiation. The hypoglossal nucleus may be used as a model of radiation-induced injury in the central nervous system, providing visual information and displaying apoptotic nuclear morphology.

## Introduction

Neuronal cell apoptosis is associated with various neurological disturbances, including radiation (1). Research concerning the molecular mechanisms of neuronal cell apoptosis following radiation has enriched the number of therapeutic strategies for protection against neuronal cell death caused by radiation (2). Mitochondria are important for the initiation and/or reinforcement of cell apoptotic pathways (3,4). During apoptosis, a key event is the release of second mitochondria-derived activator of caspase (Smac)/direct IAP binding protein with low pI (DIABLO) and cytochrome *c* (cyto *c*) from mitochondria to the cytosol (5). When the discovery of Smac by Du *et al* was first published (6), Verhagen *et al* concurrently reported on the same protein, which they named DIABLO (7). Hence, the name Smac/DIABLO is typically assigned to the molecule to credit the work of both groups. For simplicity, in the present study this molecule is referred to as Smac.

Radiation and other agents induce caspase activation fundamentally via the mitochondrial pathway, which includes the mitochondrial integration of apoptotic signals and the subsequent release of cyto *c*, Smac, Omi/HtrA2 and apoptosis-inducing factor into the cytosol (6,8). This release allows the assembly of the apoptosome. The apoptosome activates caspase-9, which subsequently induces the activation of caspase-3, -6 and -7. The effector caspases cleave their cellular specific substrates and generate the typical morphology of apoptosis (8).

The inhibitors of apoptosis protein (IAP) family prevent apoptosis by interacting with and then controlling the activities of caspase-8, -9, -3 and -7 (8). Cellular IAP-1 (c-IAP1), c-IAP2 and X-linked IAP (XIAP) are three significant members of the IAP family; XIAP is particularly significant as it has numerous domains that interact with different caspases, such as caspase-3, -7 and -9 (9,10) and its BIR2 domain inhibits caspase-7 in a non-competitive manner (11). As XIAP blocks apoptosis at the effector phase, a point where multiple signaling pathways converge, treatments targeting XIAP may prove to be effective in overcoming resistance. Smac was identified as a protein that may antagonize the inhibition of apoptosis by IAPs following its release from the mitochondria in response to apoptotic stimuli (6,7). It has been demonstrated that the domain of Smac, which interacts with IAPs is a particular NH_2_-terminal residue consisting of four amino acids, Ala-Val-Pro-Ile (12–14). Studies have shown that apoptosis-associated cyto *c* and Smac release from mitochondria occur via different mechanisms and that the release of Smac may be a key event linking the mitochondrial and death receptor pathways (15,16).

Previous studies of XIAP and Smac have mainly concentrated on tumors and cerebral ischemia reperfusion injury with less focus on radiation brain injury (17,18). Whether the interaction of XIAP and Smac affects neuronal apoptosis following brain injury induced by radiation remain unclear. It is also not known whether the expression levels of XIAP induced by radiation injury change markedly or, following irradiation, whether caspase family members are activated sequentially. Changes in the hypoglossal nucleus were investigated in rats following radiation injury, with and without caspase inhibition, to explore these unknown factors.

## Materials and methods

### Radiation model

All animal procedures were performed in a facility accredited by the Radiation Hazard Evaluation Laboratory of the Institute of Radiation Medicine of Chinese Academy of Medical Science and Peking Union Medical College (Nankai, China). All experimental procedures were performed according to the Guide for the Care and Use of Laboratory Animals published by the US National Institutes of Health (publication no. 85–23, revised 1996). Adult male Sprague-Dawley rats (weight, 250–300 g) were randomly divided into the irradiation group (IR group, n=12), the irradiation with z-VAD-fmk group (IRVAD group, n=12) or control group (con group, n=12). The irradiation of the rats in the former group was performed at room temperature using a Cs-137 γ-ray instrument (Atomic Energy of Canadian Inc., Mississauga, Canada) to administer a 4-Gy dose of radiation at a dose rate of 0.71 116 Gy/min. The animals in the control group did not receive any radiation. The study was reviewed and approved by the Institutional Animal Care and Use Committee (IACUC) of Institute of Radiation Medicine of Chinese Academy of Medical Science and Peking union Medical College (Tianjin, China).

### Intracerebroventricular administration of z-VAD-fmk

With a rat brain stereotaxic apparatus (Stoelting Co., Wood Dale, IL, USA), rats were implanted intracerebroventricularly (i.c.v.) with a cannula [anteroposterior (AP)=−2.4 mm, length, −1.4 mm; height, −3.0 mm] and osmotic micropump (Alzet^®^ micro-osmotic pump Model 1007D, Durect Corporation, CA, USA). Infusion of 2 μg z-VAD-fmk (BioVision, Mountain View, CA, USA) in 10 μl vehicle was conducted at a rate of 0.2 μg/h for 1 h. The drug vehicle was 0.5% dimethyl sulfoxide in phosphate-buffered saline (PBS). Infusions were performed at the onset of radiation administration, as previously described (19). The rats in the IRVAD group were infused with z-VAD-fmk, the other two groups were infused with vehicle. Non-irradiated controls received vehicle i.c.v. and radiation controls received z-VAD-fmk. Twenty-four hours subsequent to irradiation, the rats from each group were anesthetized with 10% chloral hydrate (30 mg/kg body weight) by intraperitoneal anesthesia.

### Immunohistology and terminal deoxynucleotidyl transferase dUTP nick end-labeling (TUNEL) staining

Rat brains were harvested and immediately frozen in 2-methylbutane at −30°C. The brainstem was cut into 12-μm thick sections with a cryostat (CM 3000; Leica, Manheim, Germany) at the level of the hypoglossal nucleus (20) and then stored at −80°C until required for further experiments. Coronal sections were air dried for 15 min, post-fixed in 10% formalin for 15 min, washed twice in PBS and then processed for immunohistology with rabbit anti-XIAP (1:1,500 dilution; Abcam, Cambridge, MA, USA). The avidin-biotin-peroxidase complex method was conducted as previously described (21). For detection of DNA fragmentation, the fluorescein-based TUNEL assay (Roche Molecular Biochemicals, Indianapolis, IN, USA) was used. TUNEL staining was conducted according to the manufacturer’s instructions. Briefly, sections were incubated for 90 min at 37°C with TUNEL reaction mixture. Positive control sections were incubated with 200 U/ml DNase I (Gibco-BRL, Carlsbad, CA, USA) for 5 min prior to fixation. Negative control sections underwent the same procedure but terminal deoxynucleotidyl transferase was omitted from the reaction buffer to evaluate nonspecific labeling. TUNEL cell counts were performed on brain sections (n=3) from the hypoglossal nuclei. TUNEL-positive cells were averaged from counts on three adjacent brain sections of a rat. Images were visualized using a Leica microscope under an excitation/emission wavelength of 500/550 nm (green), captured using an Optronics DEI-750 3-chip camera equipped with a BQ 8000 sVGA frame grabber and analyzed with Bioquant software (Bioquant Image Analysis Corporation, Nashville, TN, USA).

### Generation of cytosolic fractions

Brainstems containing the hypoglossal nucleus were collected from the rats, and cytosolic fractionation was performed as previously described (20). Briefly, the brainstem samples (6 samples per group) were homogenized in radioimmunoprecipitation assay buffer (Sigma-Aldrich Inc., St. Louis, MO, USA) containing protease inhibitors. The protein concentration of the supernatant homogenate was determined using a Bio-Rad kit (Bio-Rad, Hercules, CA, USA). Samples were then centrifuged at 2,500 × g for 15 min at 4°C to precipitate the nuclei and cellular debris. The supernatant was then centrifuged at 15,000 × g for 20 min at 4°C to remove the mitochondria. The supernatant was subsequently centrifuged at 100,000 × g for 60 min to at 4°C obtain the cytosol (supernatant).

### Western blot analysis

The protein concentration from the cytosol (supernatant) was determined spectrophotometrically from the absorbance at 595 nm (A595 nm) using the Bradford method (22). Samples (80 μg) were denatured in gel-loading buffer and separated on 15% SDS-PAGE gels, then transferred to polyvinylidene difluoride membranes and incubated with the following primary antibodies: Rabbit polyclonal anti-XIAP (1:500 dilution; Abcam), rabbit polyclonal antibody raised against Smac (1:500 dilution; Chemicon, Temecula, CA, USA), rabbit polyclonal antibody raised against cyto *c* (1:200 dilution; Santa Cruz Biotechnology, Inc., Santa Cruz, CA, USA), rabbit anti-β-actin (1:1,500 dilution; Sangon Biotech, Shanghai, China) and goat anti-rabbit IgG conjugated to horseradish peroxidase (1:800 dilution; ZSGB-BIO, Beijing, China).

### RNA extraction, cDNA synthesis and quantitative polymerase chain reaction (qPCR)

Total RNA was purified and extracted as conducted previously by Chen *et al*(23). Equal concentrations of total RNA were reverse-transcribed using Prime Script RT reagent kit (Takara Bio, Inc., Shiga, Japan) according to the manufacturer’s instructions. cDNA samples were blended with primers and SYBR Master Mix (Invitrogen Life Technologies, Carlsbad, CA, USA) in a total volume of 25 μl. All samples were assayed in triplicate using an ABI PRISM 7500 Sequence Detection system (Applied Biosystems^®^-Life Technologies, Foster City, CA, USA). The cycle threshold (CT) values for each reaction were determined and the mean was calculated using TaqMan SDS analysis software (Applied Biosystems-Life Technologies). The expression levels of target genes were calculated by the comparative CT method [fold changes=2(^−ΔΔ^CT)]. PCR primers for caspase-3, -8 and -9 and the housekeeping gene, GAPDH, were obtained from Sangon Biotech. The primer pairs used were as follows: CASP3, 5′-ATCACAGCAAAAGGAGCAGTTT-3′ (forward) and 5′-ACACCACT GTCTGTCTCAATGC-3′ (reverse); CAPS8, 5′-TAGGGACAGGAATGGAACACA-3′ (forward) and 5′-TGGGAGAGGATACAGCAGATG-3′ (reverse); CASP9, 5′-TCTGGAGGATTTGGTGATGTC-3′ (forward) and 5′-CATTTTCTTGGCAGTCAGGTC-3′ (reverse); and GAPDH, 5′-ATGACATCAAGAAGGTGGTG-3′ (forward) and 5′-CATACCAGGAAA TGAGCTTG-3′ (reverse).

### Caspase activation assay

The activities of caspase-3, -8 and -9 were analyzed using a fluorogenic caspase assay with Ac-DEVD-AFC, Ac-IETD-AFC and Ac-LEHD-AFC (BD Pharmingen, San Diego, CA, USA), respectively, as substrates (23). The results are expressed as the fold change compared with that of the control according to the previously described technique by Chen *et al*(23).

### Statistical analysis

Data are presented as the mean ± standard deviation. Data were analyzed using one-way analysis of variance with a post hoc test (multiple comparison test), which determined the significant differences between groups. P<0.05 was considered to indicate a statistically significant difference.

## Results

### Expression of XIAP and TUNEL-positive cells within the hypoglossal nuclei

**Figure 1:**
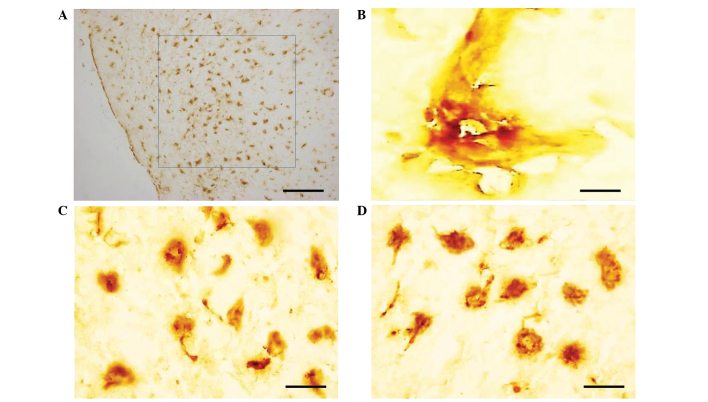
Quantification of XIAP protein expression in the hypoglossal nucleus. Brainstem sections were immunohistochemically stained with anti-XIAP antibody according to the ABC method. XIAP was positively expressed in the neurons in the hypoglossal nucleus in the (A–C) control and (D) irradiated rats. Scale bars: (A) 100 μm; (B) 5 μm; and (C,D) 20 μm.XIAP, x-linked inhibitors of apoptosis protein.

**Figure 2:**
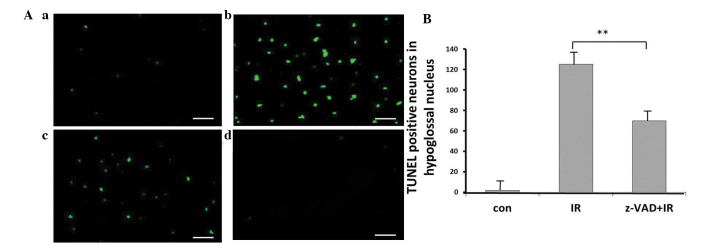
(A) TUNEL staining showed positive neurons in the hypoglossal nucleus. (a) The neurons in hypoglossal nucleus from control rats display few positive neurons (b) radiated rats remarkably become evident by a prominent growth in the number of TUNEL positive cells (c) z-VAD-fmk administration on TUNEL positive neurons following radiation remarkably decreased compared with only radiated. (d) Negative control of radiated rats without the TdT. (magnification, ×200). Scale bars: 80 μm. (B) Bar chart of the number of TUNEL-positive cells (n=6 per group). Data are expressed as the mean ± standard deviation. ^**^P<0.01. TUNEL, terminal deoxynucleotidyl transferase dUTP nick end-labeling; IR, irradiation; z-VAD, z-VAD-fmk (pan-caspase inhibitor).

XIAP was mainly expressed in the cytoplasm with positive yellow-brown staining and a high concentration of brown granules (Fig. 1A and B). In the brain tissue of the normal control group, XIAP was predominantly expressed in the perinuclear region of neurons (Fig. 1C). The levels of XIAP present in the brainstems following irradiation (Fig. 1D) were similar to those in the normal control. TUNEL-positive cells were visible mainly in the hypoglossal nuclei of the group treated with radiation alone (Fig. 2A). The number of TUNEL-positive cells detected in the control rats was low (Fig. 2B).

### Neuroprotective effects of the pan-caspase inhibitor, z-VAD-fmk, in vivo

The quantification of TUNEL-positive neurons 24 h after exposure to radiation in the hypoglossal nuclei indicated the neuroprotective effects of the pan-caspase inhibitor, z-VAD-fmk (Fig. 2). The number of TUNEL-positive neurons observed in the hypoglossal nuclei in the irradiation plus z-VAD-fmk group following irradiation was reduced compared with that of the radiation alone group (P<0.01; Fig. 2).

### Western blot analysis of XIAP, Smac and cyto c following irradiation

**Figure 3:**
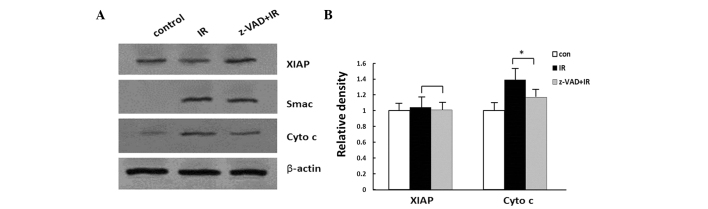
Effects of z-VAD-fmk (a pan-caspase inhibitor) on the expression levels of XIAP, Smac and cyto c. (A) Western blot analysis representative pattern from six rats. (B) Expression levels are expressed as relative density data which are the mean ± standard deviation from six rats in two independent experiments.^*^P<0.05. XIAP, X-linked inhibitors of apoptosis protein; cyto *c*, cytochrome c; IR, irradiation.

The results of the western blot analysis of XIAP, Smac and cyto *c* following irradiation are shown in Fig. 3. The presence of Smac and cyto *c* in the cytosol was observed following radiation (P<0.05). No significant changes were identified in the expression levels of XIAP following radiation (P>0.05; Fig. 3).

### Effects of z-VAD-fmk on cyto c and XIAP following irradiation

The rats were injected with z-VAD-fmk i.c.v. to investigate the effects of caspase inhibition on the expression of XIAP, as well as the release of cyto *c* and Smac. Western blot analysis demonstrated that the inhibition of caspase induced by z-VAD-fmk following radiation decreased the expression levels of cyto *c* in animals treated with z-VAD-fmk for 24 h (P<0.01; Fig. 3). By contrast, no differences in the cytoplasmic expression levels of XIAP or Smac between the z-VAD-fmk-treated animals and the vehicle-treated animals were identified following irradiation (P>0.05; Fig. 3).

### Caspase expression and activity

**Figure 4:**
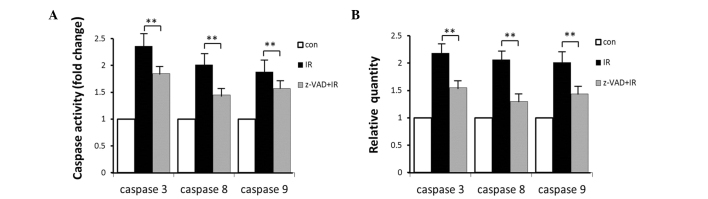
Neurons were treated with radiation or the combination of radiation and z-VAD-fmk (a pan-caspase inhibitor). (A) Activity and (B) mRNA expression levels of caspase-3, -8 and -9 were measured. ^**^P<0.01. IR, irradiation.

The effects of exposure to radiation were dependent on caspase expression and activity. The mRNA expression levels of caspase-3, -8 and -9 were measured. In the brainstem, treatment with radiation induced 2.16-, 2.06- and 2.01-fold increases in the RNA expression levels of caspase-3, -8 and -9, respectively, and increased the activities of these caspases by 2.36-, 2.01- and 1.88-fold, respectively. Combined treatment with radiation and z-VAD-fmk resulted in significant reductions in the caspase RNA expression levels of 1.55-, 1.30- and 1.44-fold and activity of 1.85-, 1.45- and 1.57-fold for caspase-3, -8 and -9, respectively (Fig. 4).

## Discussion

The IAP proteins are signal transducers that perform a diverse array of functions, which affect numerous signaling pathways and elicit multiple responses in the cell (24,25). IAPs are a family of proteins that are defined by the presence of 70 amino acids and a baculovirus IAP repeat (BIR), which was originally described as a domain utilized by viruses to compromise cell death machinery in its host (26); homologs have been identified in species ranging from baculovirus to man (25). The BIR domain is responsible for mediating protein-protein interactions, which allow certain IAPs to directly bind to and suppress caspase function to inhibit cell death (25). In addition to BIR domains, the mammalian IAPs, XIAP, c-IAP1 and c-IAP2 contain a carboxy-terminal RING domain that provides them with E3 ubiquitin ligase activity (27). In addition to their ability to conjugate ubiquitin onto target substrates, XIAP, c-IAP1 and c-IAP2 all contain a ubiquitin-associated domain (25). This motif interacts with ubiquitin chains and allows IAP proteins to participate in ubiquitin-dependent signaling pathways. c-IAP1 and c-IAP2 each contain an additional caspase activation and recruitment domain, which is thought to mediate protein-protein interactions (28). However, its function within the context of the c-IAPs remains unclear.

Smac resides within the mitochondrial intermembrane space and is subsequently released into the cytosol upon the induction of apoptosis (6). Smac and additional mitochondrial proteins, such as Omi, adenylate kinase-2, cyto *c* and apoptosis-inducing factors are released into the cytosol through permeability of the mitochondrial membrane. Proteins of the Bcl-2 family are pivotal as they may facilitate this process (29). Although mitochondrial membrane changes may result in the coincidental release of Smac and cyto *c*, the interaction of these mitochondrial proteins is also significantly interrelated, as cells deficient in cyto *c* are unable to release Smac from the mitochondria even in the presence of Bax (30). Conversely, Smac release may be necessary for the efficient release of cyto *c*(31). Furthermore, although Smac and cyto *c* release from the mitochondria occurs in the intrinsic pathway of apoptosis, complete functioning of the extrinsic pathway may require Bax-stimulated release of Smac as well. This is due to the ability of Smac to attenuate the inhibitory effects of IAPs on caspases, which may result in alterations of upstream caspases and the upregulation of extrinsic pathway receptors. Thus, although IAPs inhibit apoptosis, mitochondrial release of Smac serves to promote apoptosis by blocking the effects of IAPs on caspases.

To completely understand the role of caspases in the hypoglossal nucleus model, a cell permeable pan-caspase inhibitor, z-VAD-fmk, was used to investigate the effects of caspase blockade *in vivo*. TUNEL staining demonstrated that z-VAD-fmk reduced the numbers of TUNEL-positive cells within the hypoglossal nucleus, suggesting that intervention in the caspase cascade following radiation may have therapeutic applications. The results of the present study confirmed that inhibition of caspase by z-VAD-fmk reduced the expression levels and activation of caspase -3, -8 and -9, indicating that caspase may be a potential therapeutic target for the treatment of brain radiation injury. z-VAD-fmk reduced the appearance of cyto *c* within the cytosolic fraction following radiation. A previous study demonstrated that Smac and IAP family members are involved in regulating caspase activation (18). In the present study, western blot analysis indicated that z-VAD-fmk decreased the mitochondrial release of cyto *c*, indicating that removal of caspase activity reduced mitochondrial events in addition to caspase-3 activation. In contrast to cyto *c*, the expression of Smac was not altered by z-VAD-fmk. A previous study found that Smac is not released from the mitochondria during apoptotic signaling in cells deficient in cyto *c*, indicating that Smac release is dependent on cyto *c*(32).

The hypoglossal nucleus in the brainstem has a relatively large size and the distribution of neurons is relatively balanced, so locating the nucleus is relatively easy (Fig. 1A). Since z-VAD-fmk does not penetrate the blood-brain barrier (19), it was applied intracerebroventricularly as a bolus injection to overcome this limitation. Via the cerebrospinal fluid circulating through the fourth ventricle, the agent reaches the neurons through the process of osmosis. The hypoglossal nucleus may be used a model of radiation-induced injury in the central nervous system, providing visual information and apoptotic nuclear morphology. The hypoglossal nucleus has a large number of mitochondria (33), and therefore was an effective model for this study. In the present study, changes of the hypoglossal nucleus in an animal model established by exposure to radiation were examined. Notably, the cytosol was extracted from cells of the brainstem corresponding to the hypoglossal nucleus. In conclusion, the inhibition of caspase induced by z-VAD-fmk reduced the expression and activation of the caspase-3, -8 and -9. The present results may provide a potential theoretical basis for the therapy of brain radiation injury.
